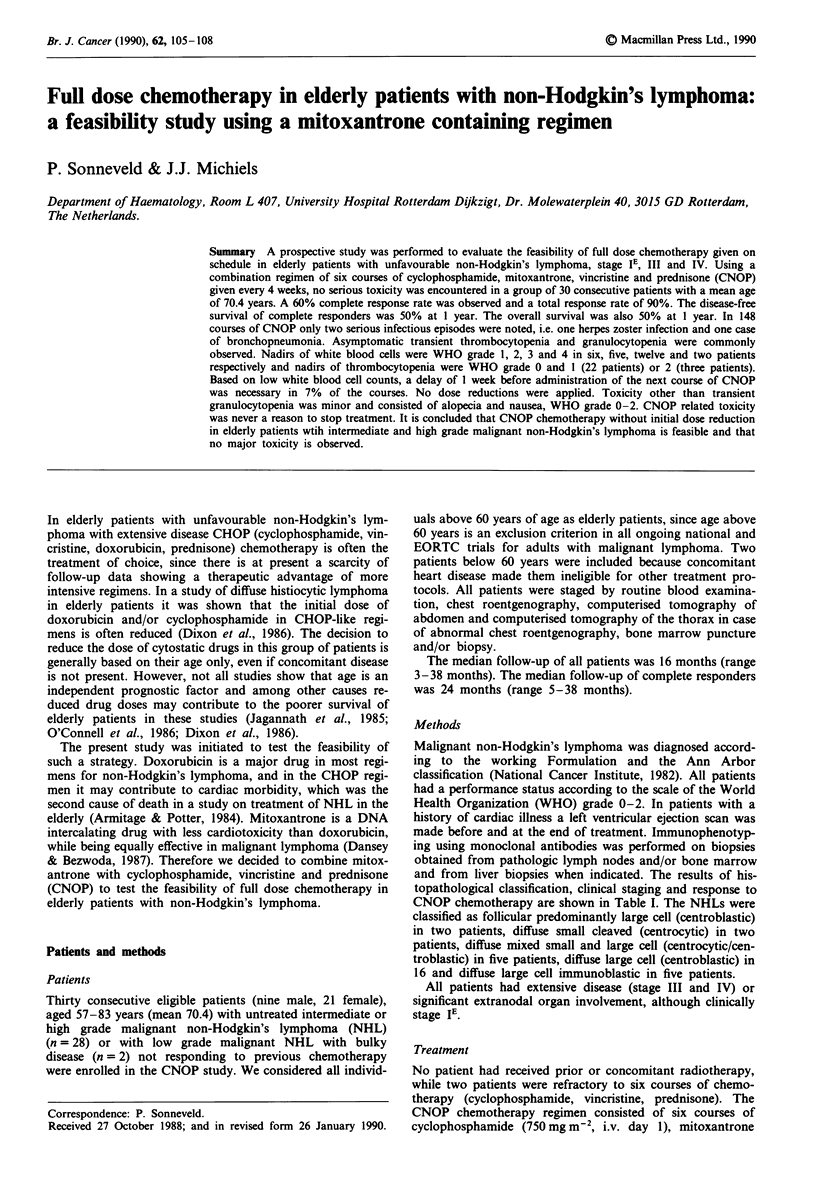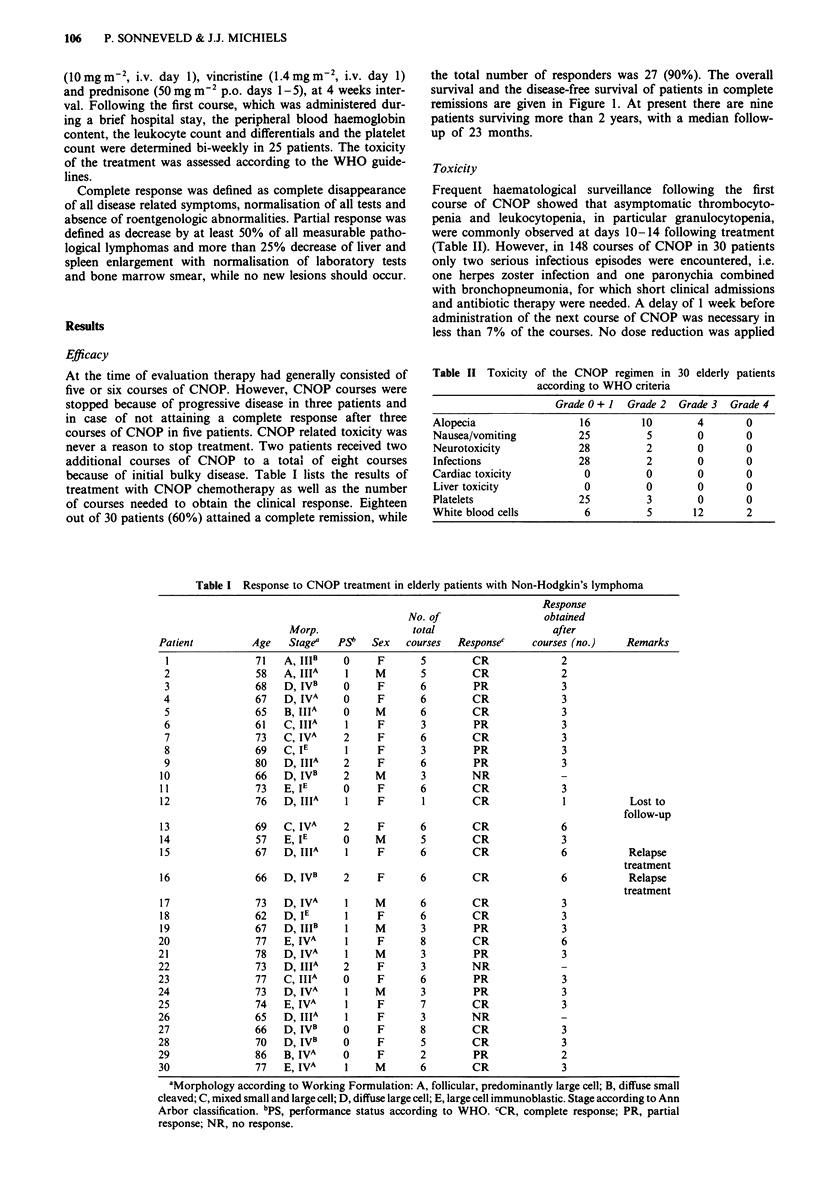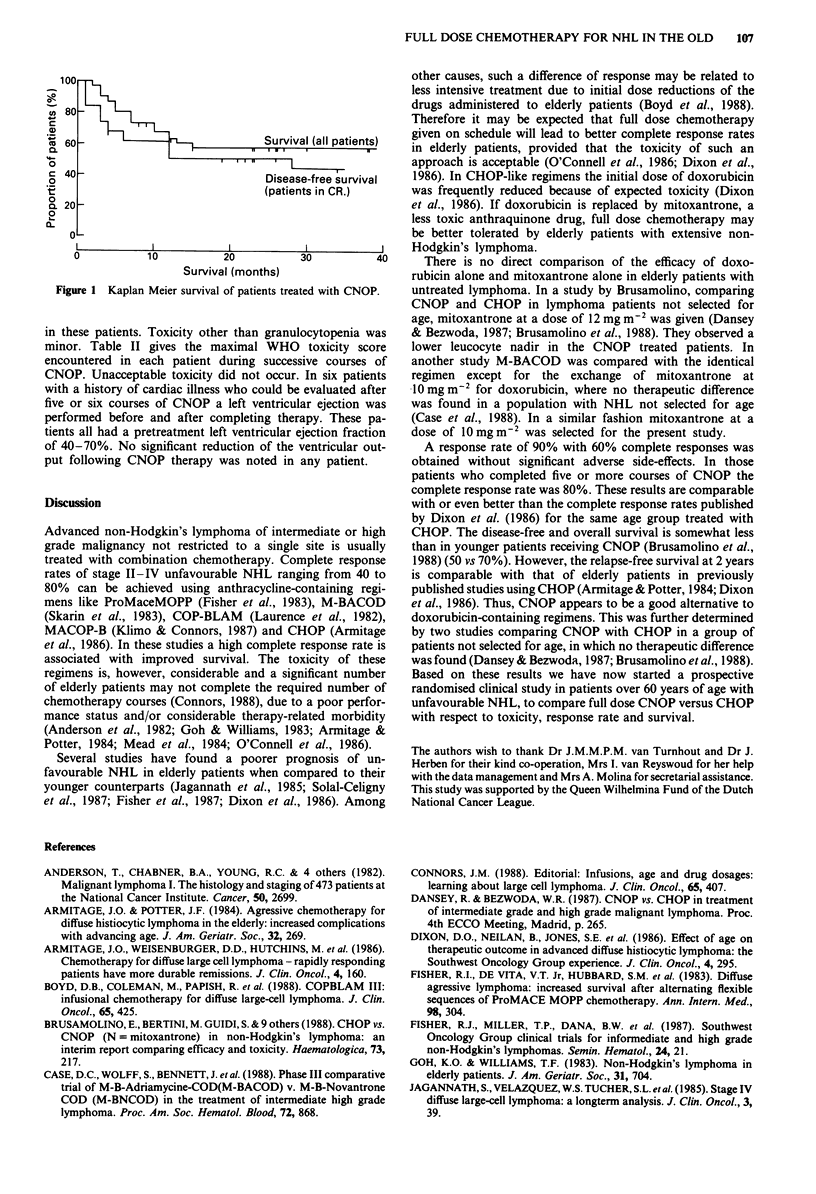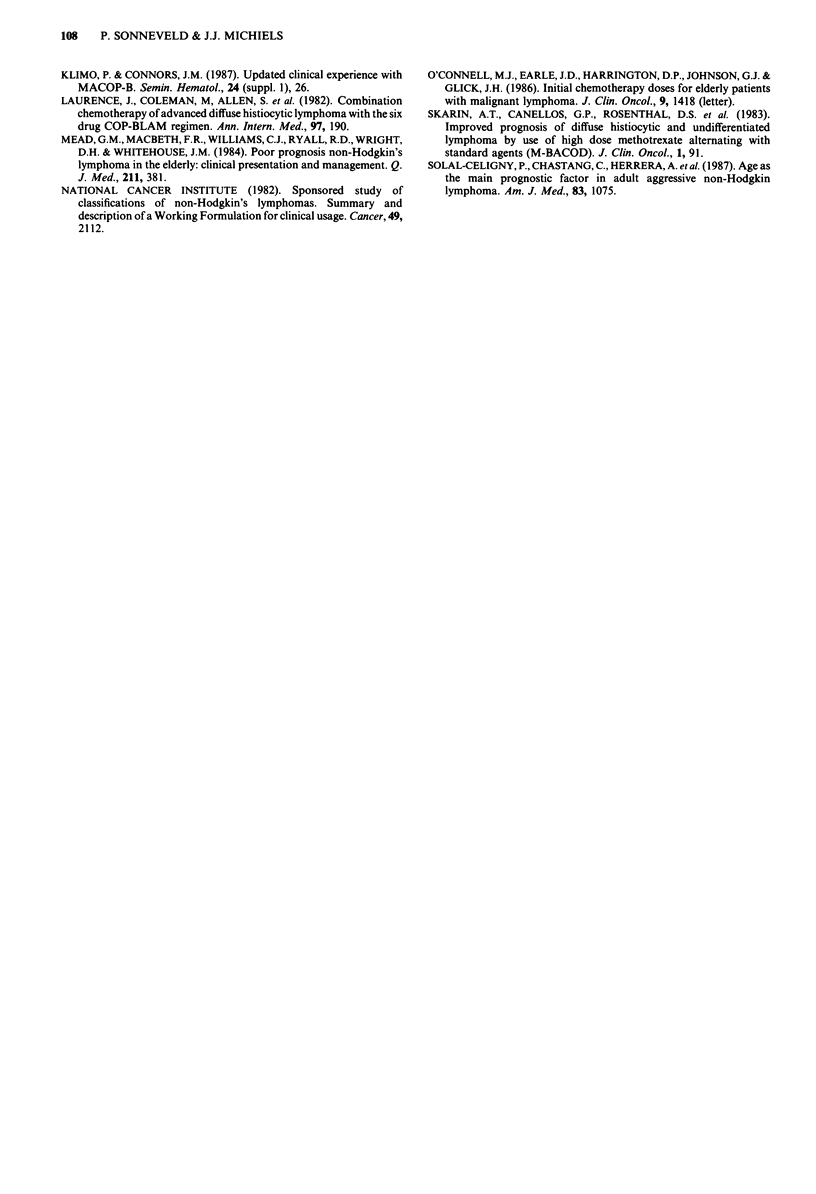# Full dose chemotherapy in elderly patients with non-Hodgkin's lymphoma: a feasibility study using a mitoxantrone containing regimen.

**DOI:** 10.1038/bjc.1990.238

**Published:** 1990-07

**Authors:** P. Sonneveld, J. J. Michiels

**Affiliations:** Department of Haematology, University Hospital Rotterdam Dijkzigt, The Netherlands.

## Abstract

A prospective study was performed to evaluate the feasibility of full dose chemotherapy given on schedule in elderly patients with unfavourable non-Hodgkin's lymphoma, stage IE, III and IV. Using a combination regimen of six courses of cyclophosphamide, mitoxantrone, vincristine and prednisone (CNOP) given every 4 weeks, no serious toxicity was encountered in a group of 30 consecutive patients with a mean age of 70.4 years. A 60% complete response rate was observed and a total response rate of 90%. The disease-free survival of complete responders was 50% at 1 year. The overall survival was also 50% at 1 year. In 148 courses of CNOP only two serious infectious episodes were noted, i.e. one herpes zoster infection and one case of bronchopneumonia. Asymptomatic transient thrombocytopenia and granulocytopenia were commonly observed. Nadirs of white blood cells were WHO grade 1, 2, 3 and 4 in six, five, twelve and two patients respectively and nadirs of thrombocytopenia were WHO grade 0 and 1 (22 patients) or 2 (three patients). Based on low white blood cell counts, a delay of 1 week before administration of the next course of CNOP was necessary in 7% of the courses. No dose reductions were applied. Toxicity other than transient granulocytopenia was minor and consisted of alopecia and nausea. WHO grade 0-2. CNOP related toxicity was never a reason to stop treatment. It is concluded that CNOP chemotherapy without initial dose reduction in elderly patients with intermediate and high grade malignant non-Hodgkin's lymphoma is feasible and that no major toxicity is observed.


*Br. J. Cancer* (1990), **62**, 105–108 © Macmillan Press Ltd., 1990

### Full dose chemotherapy in elderly patients with non-Hodgkin's lymphoma: a feasibility study using a mitoxantrone containing regimen

P. Sonneveld & J.J. Michiels

*Department of Haematology, Room L 407, University Hospital Rotterdam Dijkzigt, Dr. Molewaterplein 40, 3015 GD Rotterdam, The Netherlands.*

**Summary** A prospective study was performed to evaluate the feasibility of full dose chemotherapy given on schedule in elderly patients with unfavourable non-Hodgkin's lymphoma, stage $I^E$, III and IV. Using a combination regimen of six courses of cyclophosphamide, mitoxantrone, vincristine and prednisone (CNOP) given every 4 weeks, no serious toxicity was encountered in a group of 30 consecutive patients with a mean age of 70.4 years. A 60% complete response rate was observed and a total response rate of 90%. The disease-free survival of complete responders was 50% at 1 year. The overall survival was also 50% at 1 year. In 148 courses of CNOP only two serious infectious episodes were noted, i.e. one herpes zoster infection and one case of bronchopneumonia. Asymptomatic transient thrombocytopenia and granulocytopenia were commonly observed. Nadirs of white blood cells were WHO grade 1, 2, 3 and 4 in six, five, twelve and two patients respectively and nadirs of thrombocytopenia were WHO grade 0 and 1 (22 patients) or 2 (three patients). Based on low white blood cell counts, a delay of 1 week before administration of the next course of CNOP was necessary in 7% of the courses. No dose reductions were applied. Toxicity other than transient granulocytopenia was minor and consisted of alopecia and nausea, WHO grade 0–2. CNOP related toxicity was never a reason to stop treatment. It is concluded that CNOP chemotherapy without initial dose reduction in elderly patients wtih intermediate and high grade malignant non-Hodgkin's lymphoma is feasible and that no major toxicity is observed.

In elderly patients with unfavourable non-Hodgkin's lymphoma with extensive disease CHOP (cyclophosphamide, vincristine, doxorubicin, prednisone) chemotherapy is often the treatment of choice, since there is at present a scarcity of follow-up data showing a therapeutic advantage of more intensive regimens. In a study of diffuse histiocytic lymphoma in elderly patients it was shown that the initial dose of doxorubicin and/or cyclophosphamide in CHOP-like regimens is often reduced (Dixon *et al.*, 1986). The decision to reduce the dose of cytostatic drugs in this group of patients is generally based on their age only, even if concomitant disease is not present. However, not all studies show that age is an independent prognostic factor and among other causes reduced drug doses may contribute to the poorer survival of elderly patients in these studies (Jagannath *et al.*, 1985; O'Connell *et al.*, 1986; Dixon *et al.*, 1986).

The present study was initiated to test the feasibility of such a strategy. Doxorubicin is a major drug in most regimens for non-Hodgkin's lymphoma, and in the CHOP regimen it may contribute to cardiac morbidity, which was the second cause of death in a study on treatment of NHL in the elderly (Armitage & Potter, 1984). Mitoxantrone is a DNA intercalating drug with less cardiotoxicity than doxorubicin, while being equally effective in malignant lymphoma (Dansey & Bezwoda, 1987). Therefore we decided to combine mitoxantrone with cyclophosphamide, vincristine and prednisone (CNOP) to test the feasibility of full dose chemotherapy in elderly patients with non-Hodgkin's lymphoma.

### Patients and methods

### *Patients*

Thirty consecutive eligible patients (nine male, 21 female), aged 57–83 years (mean 70.4) with untreated intermediate or high grade malignant non-Hodgkin's lymphoma (NHL) ($n = 28$) or with low grade malignant NHL with bulky disease ($n = 2$) not responding to previous chemotherapy were enrolled in the CNOP study. We considered all individuals above 60 years of age as elderly patients, since age above 60 years is an exclusion criterion in all ongoing national and EORTC trials for adults with malignant lymphoma. Two patients below 60 years were included because concomitant heart disease made them ineligible for other treatment protocols. All patients were staged by routine blood examination, chest roentgenography, computerised tomography of abdomen and computerised tomography of the thorax in case of abnormal chest roentgenography, bone marrow puncture and/or biopsy.

The median follow-up of all patients was 16 months (range 3–38 months). The median follow-up of complete responders was 24 months (range 5–38 months).

### *Methods*

Malignant non-Hodgkin's lymphoma was diagnosed according to the working Formulation and the Ann Arbor classification (National Cancer Institute, 1982). All patients had a performance status according to the scale of the World Health Organization (WHO) grade 0–2. In patients with a history of cardiac illness a left ventricular ejection scan was made before and at the end of treatment. Immunophenotyping using monoclonal antibodies was performed on biopsies obtained from pathologic lymph nodes and/or bone marrow and from liver biopsies when indicated. The results of histopathological classification, clinical staging and response to CNOP chemotherapy are shown in Table I. The NHLs were classified as follicular predominantly large cell (centroblastic) in two patients, diffuse small cleaved (centrocytic) in two patients, diffuse mixed small and large cell (centrocytic/centroblastic) in five patients, diffuse large cell (centroblastic) in 16 and diffuse large cell immunoblastic in five patients.

All patients had extensive disease (stage III and IV) or significant extranodal organ involvement, although clinically stage $I^E$.

### *Treatment*

No patient had received prior or concomitant radiotherapy, while two patients were refractory to six courses of chemotherapy (cyclophosphamide, vincristine, prednisone). The CNOP chemotherapy regimen consisted of six courses of cyclophosphamide (750 mg $m^{-2}$, i.v. day 1), mitoxantrone

Correspondence: P. Sonneveld.
Received 27 October 1988; and in revised form 26 January 1990.

(10 mg m$^{-2}$, i.v. day 1), vincristine (1.4 mg m$^{-2}$, i.v. day 1) and prednisone (50 mg m$^{-2}$ p.o. days 1–5), at 4 weeks interval. Following the first course, which was administered during a brief hospital stay, the peripheral blood haemoglobin content, the leukocyte count and differentials and the platelet count were determined bi-weekly in 25 patients. The toxicity of the treatment was assessed according to the WHO guidelines.

Complete response was defined as complete disappearance of all disease related symptoms, normalisation of all tests and absence of roentgenologic abnormalities. Partial response was defined as decrease by at least 50% of all measurable pathological lymphomas and more than 25% decrease of liver and spleen enlargement with normalisation of laboratory tests and bone marrow smear, while no new lesions should occur.

### Results

### *Efficacy*

At the time of evaluation therapy had generally consisted of five or six courses of CNOP. However, CNOP courses were stopped because of progressive disease in three patients and in case of not attaining a complete response after three courses of CNOP in five patients. CNOP related toxicity was never a reason to stop treatment. Two patients received two additional courses of CNOP to a total of eight courses because of initial bulky disease. Table I lists the results of treatment with CNOP chemotherapy as well as the number of courses needed to obtain the clinical response. Eighteen out of 30 patients (60%) attained a complete remission, while the total number of responders was 27 (90%). The overall survival and the disease-free survival of patients in complete remissions are given in Figure 1. At present there are nine patients surviving more than 2 years, with a median follow-up of 23 months.

### *Toxicity*

Frequent haematological surveillance following the first course of CNOP showed that asymptomatic thrombocytopenia and leukocytopenia, in particular granulocytopenia, were commonly observed at days 10–14 following treatment (Table II). However, in 148 courses of CNOP in 30 patients only two serious infectious episodes were encountered, i.e. one herpes zoster infection and one paronychia combined with bronchopneumonia, for which short clinical admissions and antibiotic therapy were needed. A delay of 1 week before administration of the next course of CNOP was necessary in less than 7% of the courses. No dose reduction was applied

**Table II** Toxicity of the CNOP regimen in 30 elderly patients according to WHO criteria

| | *Grade 0 + 1* | *Grade 2* | *Grade 3* | *Grade 4* |
|---|---|---|---|---|
| Alopecia | 16 | 10 | 4 | 0 |
| Nausea/vomiting | 25 | 5 | 0 | 0 |
| Neurotoxicity | 28 | 2 | 0 | 0 |
| Infections | 28 | 2 | 0 | 0 |
| Cardiac toxicity | 0 | 0 | 0 | 0 |
| Liver toxicity | 0 | 0 | 0 | 0 |
| Platelets | 25 | 3 | 0 | 0 |
| White blood cells | 6 | 5 | 12 | 2 |

**Table I** Response to CNOP treatment in elderly patients with Non-Hodgkin's lymphoma

| *Patient* | *Age* | *Morp. Stage*[a] | *PS*[b] | *Sex* | *No. of total courses* | *Response*[c] | *Response obtained after courses (no.)* | *Remarks* |
|---|---|---|---|---|---|---|---|---|
| 1 | 71 | A, III$^B$ | 0 | F | 5 | CR | 2 | |
| 2 | 58 | A, III$^A$ | 1 | M | 5 | CR | 2 | |
| 3 | 68 | D, IV$^B$ | 0 | F | 6 | PR | 3 | |
| 4 | 67 | D, IV$^A$ | 0 | F | 6 | CR | 3 | |
| 5 | 65 | B, III$^A$ | 0 | M | 6 | CR | 3 | |
| 6 | 61 | C, III$^A$ | 1 | F | 3 | PR | 3 | |
| 7 | 73 | C, IV$^A$ | 2 | F | 6 | CR | 3 | |
| 8 | 69 | C, I$^E$ | 1 | F | 3 | PR | 3 | |
| 9 | 80 | D, III$^A$ | 2 | F | 6 | PR | 3 | |
| 10 | 66 | D, IV$^B$ | 2 | M | 3 | NR | – | |
| 11 | 73 | E, I$^E$ | 0 | F | 6 | CR | 3 | |
| 12 | 76 | D, III$^A$ | 1 | F | 1 | CR | 1 | Lost to follow-up |
| 13 | 69 | C, IV$^A$ | 2 | F | 6 | CR | 6 | |
| 14 | 57 | E, I$^E$ | 0 | M | 5 | CR | 3 | |
| 15 | 67 | D, III$^A$ | 1 | F | 6 | CR | 6 | Relapse treatment |
| 16 | 66 | D, IV$^B$ | 2 | F | 6 | CR | 6 | Relapse treatment |
| 17 | 73 | D, IV$^A$ | 1 | M | 6 | CR | 3 | |
| 18 | 62 | D, I$^E$ | 1 | F | 6 | CR | 3 | |
| 19 | 67 | D, III$^B$ | 1 | M | 3 | PR | 3 | |
| 20 | 77 | E, IV$^A$ | 1 | F | 8 | CR | 6 | |
| 21 | 78 | D, IV$^A$ | 1 | M | 3 | PR | 3 | |
| 22 | 73 | D, III$^A$ | 2 | F | 3 | NR | – | |
| 23 | 77 | C, III$^A$ | 0 | F | 6 | PR | 3 | |
| 24 | 73 | D, IV$^A$ | 1 | M | 3 | PR | 3 | |
| 25 | 74 | E, IV$^A$ | 1 | F | 7 | CR | 3 | |
| 26 | 65 | D, III$^A$ | 1 | F | 3 | NR | – | |
| 27 | 66 | D, IV$^B$ | 0 | F | 8 | CR | 3 | |
| 28 | 70 | D, IV$^B$ | 0 | F | 5 | CR | 3 | |
| 29 | 86 | B, IV$^A$ | 0 | F | 2 | PR | 2 | |
| 30 | 77 | E, IV$^A$ | 1 | M | 6 | CR | 3 | |

[a]Morphology according to Working Formulation: A, follicular, predominantly large cell; B, diffuse small cleaved; C, mixed small and large cell; D, diffuse large cell; E, large cell immunoblastic. Stage according to Ann Arbor classification. [b]PS, performance status according to WHO. [c]CR, complete response; PR, partial response; NR, no response.

**Figure 1** Kaplan Meier survival of patients treated with CNOP.

in these patients. Toxicity other than granulocytopenia was minor. Table II gives the maximal WHO toxicity score encountered in each patient during successive courses of CNOP. Unacceptable toxicity did not occur. In six patients with a history of cardiac illness who could be evaluated after five or six courses of CNOP a left ventricular ejection was performed before and after completing therapy. These patients all had a pretreatment left ventricular ejection fraction of 40–70%. No significant reduction of the ventricular output following CNOP therapy was noted in any patient.

### Discussion

Advanced non-Hodgkin's lymphoma of intermediate or high grade malignancy not restricted to a single site is usually treated with combination chemotherapy. Complete response rates of stage II–IV unfavourable NHL ranging from 40 to 80% can be achieved using anthracycline-containing regimens like ProMaceMOPP (Fisher *et al.*, 1983), M-BACOD (Skarin *et al.*, 1983), COP-BLAM (Laurence *et al.*, 1982), MACOP-B (Klimo & Connors, 1987) and CHOP (Armitage *et al.*, 1986). In these studies a high complete response rate is associated with improved survival. The toxicity of these regimens is, however, considerable and a significant number of elderly patients may not complete the required number of chemotherapy courses (Connors, 1988), due to a poor performance status and/or considerable therapy-related morbidity (Anderson *et al.*, 1982; Goh & Williams, 1983; Armitage & Potter, 1984; Mead *et al.*, 1984; O'Connell *et al.*, 1986).

Several studies have found a poorer prognosis of unfavourable NHL in elderly patients when compared to their younger counterparts (Jagannath *et al.*, 1985; Solal-Celigny *et al.*, 1987; Fisher *et al.*, 1987; Dixon *et al.*, 1986). Among other causes, such a difference of response may be related to less intensive treatment due to initial dose reductions of the drugs administered to elderly patients (Boyd *et al.*, 1988). Therefore it may be expected that full dose chemotherapy given on schedule will lead to better complete response rates in elderly patients, provided that the toxicity of such an approach is acceptable (O'Connell *et al.*, 1986; Dixon *et al.*, 1986). In CHOP-like regimens the initial dose of doxorubicin was frequently reduced because of expected toxicity (Dixon *et al.*, 1986). If doxorubicin is replaced by mitoxantrone, a less toxic anthraquinone drug, full dose chemotherapy may be better tolerated by elderly patients with extensive non-Hodgkin's lymphoma.

There is no direct comparison of the efficacy of doxorubicin alone and mitoxantrone alone in elderly patients with untreated lymphoma. In a study by Brusamolino, comparing CNOP and CHOP in lymphoma patients not selected for age, mitoxantrone at a dose of 12 mg $m^{-2}$ was given (Dansey & Bezwoda, 1987; Brusamolino *et al.*, 1988). They observed a lower leucocyte nadir in the CNOP treated patients. In another study M-BACOD was compared with the identical regimen except for the exchange of mitoxantrone at 10 mg $m^{-2}$ for doxorubicin, where no therapeutic difference was found in a population with NHL not selected for age (Case *et al.*, 1988). In a similar fashion mitoxantrone at a dose of 10 mg $m^{-2}$ was selected for the present study.

A response rate of 90% with 60% complete responses was obtained without significant adverse side-effects. In those patients who completed five or more courses of CNOP the complete response rate was 80%. These results are comparable with or even better than the complete response rates published by Dixon *et al.* (1986) for the same age group treated with CHOP. The disease-free and overall survival is somewhat less than in younger patients receiving CNOP (Brusamolino *et al.*, 1988) (50 *vs* 70%). However, the relapse-free survival at 2 years is comparable with that of elderly patients in previously published studies using CHOP (Armitage & Potter, 1984; Dixon *et al.*, 1986). Thus, CNOP appears to be a good alternative to doxorubicin-containing regimens. This was further determined by two studies comparing CNOP with CHOP in a group of patients not selected for age, in which no therapeutic difference was found (Dansey & Bezwoda, 1987; Brusamolino *et al.*, 1988). Based on these results we have now started a prospective randomised clinical study in patients over 60 years of age with unfavourable NHL, to compare full dose CNOP versus CHOP with respect to toxicity, response rate and survival.

The authors wish to thank Dr J.M.M.P.M. van Turnhout and Dr J. Herben for their kind co-operation, Mrs I. van Reyswoud for her help with the data management and Mrs A. Molina for secretarial assistance. This study was supported by the Queen Wilhelmina Fund of the Dutch National Cancer League.

### References

ANDERSON, T., CHABNER, B.A., YOUNG, R.C. & 4 others (1982). Malignant lymphoma I. The histology and staging of 473 patients at the National Cancer Institute. *Cancer*, **50**, 2699.

ARMITAGE, J.O. & POTTER, J.F. (1984). Agressive chemotherapy for diffuse histiocytic lymphoma in the elderly: increased complications with advancing age. *J. Am. Geriatr. Soc.*, **32**, 269.

ARMITAGE, J.O., WEISENBURGER, D.D., HUTCHINS, M. *et al.* (1986). Chemotherapy for diffuse large cell lymphoma – rapidly responding patients have more durable remissions. *J. Clin. Oncol.*, **4**, 160.

BOYD, D.B., COLEMAN, M., PAPISH, R. *et al.* (1988). COPBLAM III: infusional chemotherapy for diffuse large-cell lymphoma. *J. Clin. Oncol.*, **65**, 425.

BRUSAMOLINO, E., BERTINI, M. GUIDI, S. & 9 others (1988). CHOP *vs.* CNOP (N = mitoxantrone) in non-Hodgkin's lymphoma: an interim report comparing efficacy and toxicity. *Haematologica*, **73**, 217.

CASE, D.C., WOLFF, S., BENNETT, J. *et al.* (1988). Phase III comparative trial of M-B-Adriamycine-COD(M-BACOD) v. M-B-Novantrone COD (M-BNCOD) in the treatment of intermediate high grade lymphoma. *Proc. Am. Soc. Hematol. Blood*, **72**, 868.

CONNORS, J.M. (1988). Editorial: Infusions, age and drug dosages: learning about large cell lymphoma. *J. Clin. Oncol.*, **65**, 407.

DANSEY, R. & BEZWODA, W.R. (1987). CNOP *vs.* CHOP in treatment of intermediate grade and high grade malignant lymphoma. Proc. 4th ECCO Meeting, Madrid, p. 265.

DIXON, D.O., NEILAN, B., JONES, S.E. *et al.* (1986). Effect of age on therapeutic outcome in advanced diffuse histiocytic lymphoma: the Southwest Oncology Group experience. *J. Clin. Oncol.*, **4**, 295.

FISHER, R.I., DE VITA, V.T. Jr, HUBBARD, S.M. *et al.* (1983). Diffuse agressive lymphoma: increased survival after alternating flexible sequences of ProMACE MOPP chemotherapy. *Ann. Intern. Med.*, **98**, 304.

FISHER, R.J., MILLER, T.P., DANA, B.W. *et al.* (1987). Southwest Oncology Group clinical trials for informediate and high grade non-Hodgkin's lymphomas. *Semin. Hematol.*, **24**, 21.

GOH, K.O. & WILLIAMS, T.F. (1983). Non-Hodgkin's lymphoma in elderly patients. *J. Am. Geriatr. Soc.*, **31**, 704.

JAGANNATH, S., VELAZQUEZ, W.S. TUCHER, S.L. *et al.* (1985). Stage IV diffuse large-cell lymphoma: a longterm analysis. *J. Clin. Oncol.*, **3**, 39.

KLIMO, P. & CONNORS, J.M. (1987). Updated clinical experience with MACOP-B. *Semin. Hematol.*, **24** (suppl. 1), 26.

LAURENCE, J., COLEMAN, M, ALLEN, S. *et al.* (1982). Combination chemotherapy of advanced diffuse histiocytic lymphoma with the six drug COP-BLAM regimen. *Ann. Intern. Med.*, **97**, 190.

MEAD, G.M., MACBETH, F.R., WILLIAMS, C.J., RYALL, R.D., WRIGHT, D.H. & WHITEHOUSE, J.M. (1984). Poor prognosis non-Hodgkin's lymphoma in the elderly: clinical presentation and management. *Q. J. Med.*, **211**, 381.

NATIONAL CANCER INSTITUTE (1982). Sponsored study of classifications of non-Hodgkin's lymphomas. Summary and description of a Working Formulation for clinical usage. *Cancer*, **49**, 2112.

O'CONNELL, M.J., EARLE, J.D., HARRINGTON, D.P., JOHNSON, G.J. & GLICK, J.H. (1986). Initial chemotherapy doses for elderly patients with malignant lymphoma. *J. Clin. Oncol.*, **9**, 1418 (letter).

SKARIN, A.T., CANELLOS, G.P., ROSENTHAL, D.S. *et al.* (1983). Improved prognosis of diffuse histiocytic and undifferentiated lymphoma by use of high dose methotrexate alternating with standard agents (M-BACOD). *J. Clin. Oncol.*, **1**, 91.

SOLAL-CELIGNY, P., CHASTANG, C., HERRERA, A. *et al.* (1987). Age as the main prognostic factor in adult aggressive non-Hodgkin lymphoma. *Am. J. Med.*, **83**, 1075.